# Hip adduction and abduction strength in youth male soccer and basketball players with and without groin pain in the past year

**DOI:** 10.1371/journal.pone.0275650

**Published:** 2022-10-05

**Authors:** Jan Marušič, Nejc Šarabon

**Affiliations:** 1 Faculty of Health Sciences, University of Primorska, Izola, Slovenia; 2 Science to Practice, Ltd., Laboratory for Motor Control and Motor Behavior, Ljubljana, Slovenia; Universita degli Studi di Verona, ITALY

## Abstract

The objectives of this study were to 1) assess the differences between youth soccer and basketball players with and without past year groin pain (GP) in hip adduction and abduction strength and several training characteristics (age at the start of regular training, weekly training frequency, warm-up and training duration, use of stretching and/or stabilisation exercises during warm-up, use of resistance training); 2) present strength reference values for youth soccer and basketball players. 227 players participated (age 16.9 ± 1.4 years; height 184.2 ± 8.5 cm; mass 75.5 ± 11.9 kg). Hip adduction and abduction strength was measured in supine position (hip, knee and ankle in neutral position) using a MuscleBoard dynamometer. Interlimb asymmetries and hip adduction:abduction ratios were calculated. Past year GP and training characteristics were assessed with a retrospective questionnaire. 11.9% of players reported past year GP (16.9% in soccer and 6.4% in basketball). The only significant difference between the past year GP and the control groups was found in the age of the players at the start of regular training (7.2 ± 1.8 years for the GP group vs. 8.5 ± 2.6 years for the control group). Additionally, soccer players without past year GP have significantly higher hip adduction strength (1.1 ± 0.2 Nm/kg vs. 1.0 ± 0.2 Nm/kg) and adduction:abduction strength ratio (1.10 ± 0.18 vs. 1.03 ± 0.16) compared to basketball players. Our results show that hip adduction and abduction strength, interlimb asymmetry and hip adduction:abduction ratio do not differentiate between players with and without past year GP (p = 0.29–0.90), which means that their adduction or abduction strength can be analysed regardless of the GP presence in the past year. Additionally, players with past year GP started regularly training at significantly lower age, which could indicate the problematic nature of early/premature sports specialisation.

## Introduction

Groin pain is a common and problematic phenomenon in sports involving kicking, jumping, reaching, rapid accelerations, sudden changes of direction, pivoting, or skating [1–3]. In soccer, basketball, and hockey, groin pain is among the three most common injuries [1, 4, 5]. Specifically, a 15-year prospective study involving 47 elite European soccer teams and 3055 players shows that groin pain accounts for 14% of all injuries, and affects ~20% of soccer players each season [6]. It seems that the chronic type of groin pain in soccer seems to be more common (73%) than acute (27%) and results in a longer absence (35 days) from competition [7, 8].

The most commonly identified risk factors for groin pain occurrence are the previous episode of groin pain and reduced hip adduction (HADD) strength [9], as well as reduced HADD to hip abduction (HABD) strength ratio [10, 11]. Several methods of measuring strength have demonstrated an association between poorer HADD strength and groin pain in adult soccer players: the most commonly used hand-held dynamometry (e.g. MicroFet dynamometer) [12, 13] and newer, more sophisticated dynamometers such as ForceFrame [14] and MuscleBoard [15]. These studies found that adult soccer players with current and future groin pain have poorer HADD strength. However, little is known about whether previous groin pain has long-lasting effect on HADD strength. Esteve et al. [16] showed that adult soccer players with past-season groin pain lasting longer than 6 weeks showed 11.5–15.3% lower values of HADD strength compared with those without past-season groin pain (players with groin pain at the time of measurement were excluded from the study sample). In contrast, DeLang et al. [17] reported that past-season groin pain in youth soccer players did not have an effect on HADD strength (only players with time-loss hip or groin injury were excluded). In addition to reduced absolute strength, one study has also highlighted inter-limb asymmetries (ILA) as a possible risk factor for a groin pain occurrence in adult soccer players [15]. However, it has not yet been verified whether ILA in HADD strength is associated with groin pain in youth soccer or basketball players. 10–15% of ILA in HADD strength represents a minimal clinically relevant difference and is considered as one of the clinical milestone for return to play in athletes with groin pain [12, 18].

To the best of our knowledge, only one study to date has examined the effects of a past year groin pain on HADD strength in youth soccer players [17], and none has examined youth basketball players. The effects of past year groin pain in youth soccer or basketball players on the HADD:HABD strength ratio, ILA in HADD or HABD strength have not yet been examined. Furthermore, reference values for HADD and HABD strength, and HADD:HABD strength ratio for youth soccer and basketball players are not yet established. Additionally, the differences in these strength parameters between youth soccer and basketball players are not yet known. In the fields of injury prevention, rehabilitation and assessment of physical performance, it would be useful to know whether different demands of sport (e.g., more frequent lateral shuffling and the absence of kicking in basketball compared to soccer) [21] can cause differences in hip strength outcomes and whether these differences are (already) apparent in youth players. Therefore, the aim of this study was to investigate the differences between (a) a group that reported groin pain over the past 12 months and a control group; (b) a group of soccer and a group of basketball players without groin pain in the past 12 months in: (i) strength variables (HADD and HABD strength, HADD:HABD ratio, ILA in HABD and HADD strength); (ii) different variables regarding sports training in the past 12 months (e.g., weekly training frequency, training duration, warm-up duration, etc.). Additional purpose was to assess the impact of 5 predictors (HABD and HADD strength, ILA in HABD and HADD strength, HADD:HABD strength ratio) on the likelihood that participants had chronic groin pain in the past 12 months. The final aim of the study was to present reference values for HADD and HABD strength, and HADD:HABD ratio for youth soccer and basketball players. We hypothesized that (i) there would be statistically significant differences between the group reporting groin pain in the last 12 months and the control group in hip strength variables; (ii) soccer players would have statistically significantly higher HADD strength and HADD:HABD strength ratio compared to basketball players.

## Methods

### Participants

A convenience sample of 227 male youth athletes from the highest Slovenian football and basketball leagues (109 basketball players, 118 soccer players) was used in the study. Participants who had been training regularly for at least one year (mean 8.6 ± 2.8 years) were recruited from local and regional soccer and basketball clubs with the help of the Olympic Committee of Slovenia and Slovenian sport federations. The exclusion criterion for the strength measurements was any pain/injury that prevented the players from achieving a true maximal contraction in HABD or HADD. Athletes were assigned to the groin pain group or the control group based on a retrospective questionnaire about the presence of chronic pain in the groin area in the past 12 months (questionnaire described below). All participants were informed of the purpose and content of the study and provided written informed consent prior to participation. In the case of underage participants (under 18 years of age), parents or legal guardians were also thoroughly informed and signed the consent form. The individual shown in this manuscript (Fig 2) gave written informed consent (as outlined in PLOS consent form). The study was approved by the National Medical Ethics Committee (0120-690/2017/8) and conducted according to the Declaration of Helsinki.

### Study protocol

**Fig 1:**
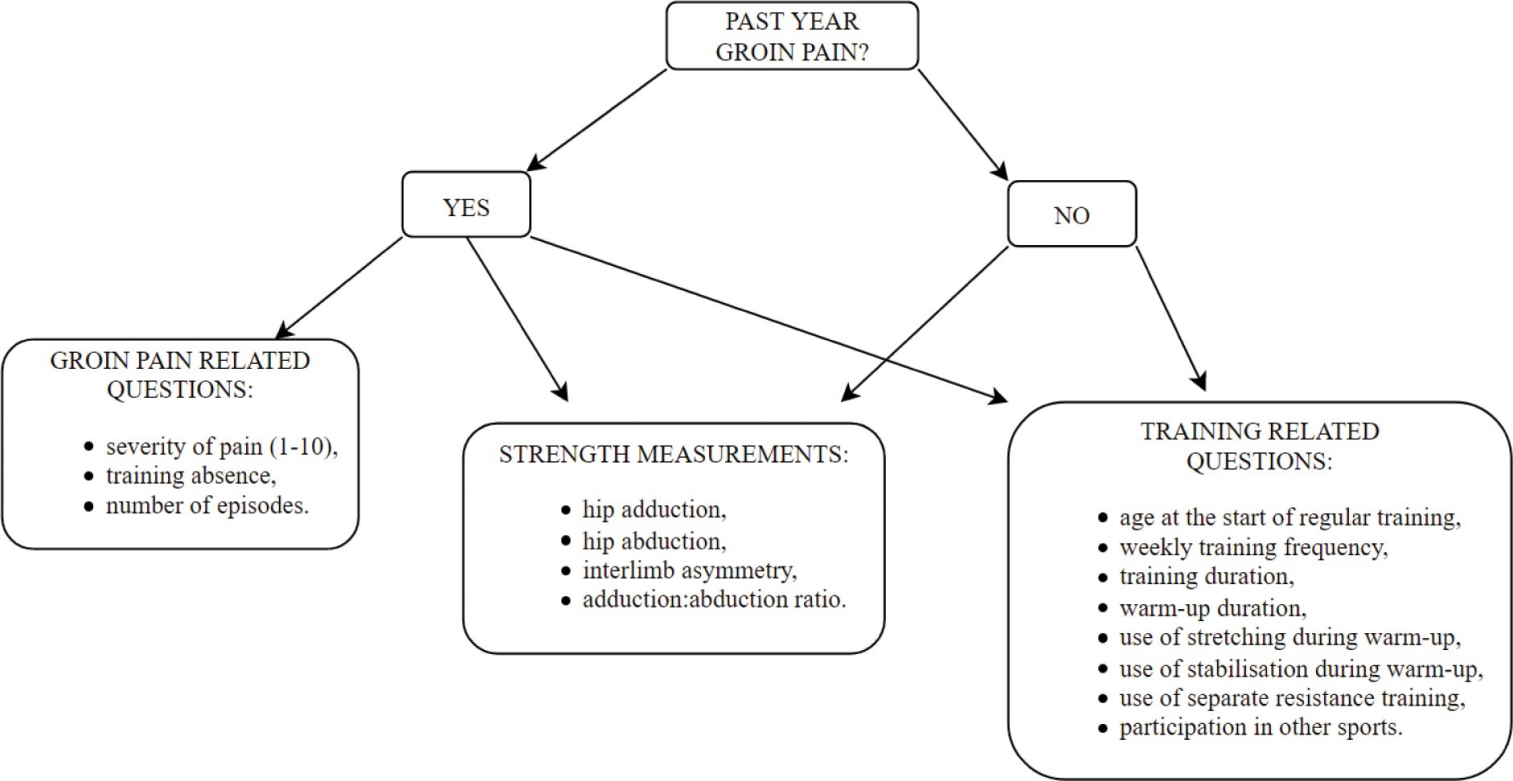
Flowchart of collected outcome variables according to the allocation of players with or without groin pain in the past year.

Prior to strength measurements, participants completed a 12-month chronic groin pain recall questionnaire, which was developed based on existing questionnaires [19]. Chronic groin pain was defined as pain that occurs gradually (does not have a clear onset as an acute injury) or occurs repeatedly (2 or more times in the past 12 months) and is located at the hip joint, adjacent soft tissues or at the junction between the antero-medial part of the thigh (this includes the proximal part of the hip adductors) and the lower abdomen [7]. Participants with groin pain additionally indicated how severe the pain was on a scale of 1 to 10 (1 indicates very mild pain and 10 extremely severe pain), whether they were absent from training due to the pain, how long the pain lasted, and whether the pain (episode) occurred more than once. All players provided basic information about their training in the past 12 months (age at the start of regular training, weekly training frequency, training duration, warm-up duration, whether they performed stretching and/or stabilisation exercises during warm-up, whether they performed separate resistance training), and about their previous sports activities (whether they played other sports before their main (current) sport and for how long). All data on groin pain and training characteristics were collected using a single self-reported recall questionnaire. An overview of all outcome variables is presented in Fig 1.

**Fig 2:**
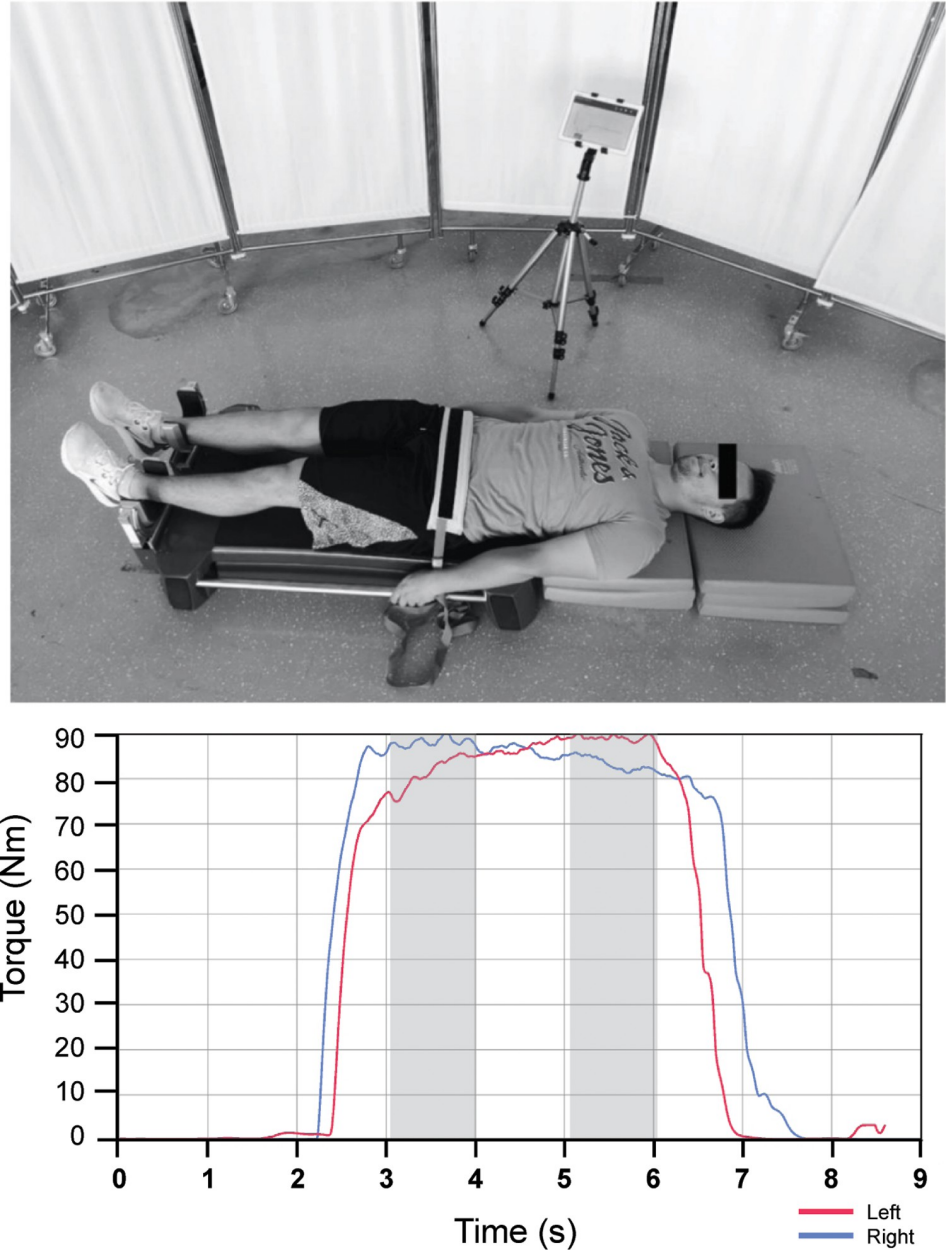
Hip adduction and abduction strength testing position (top part) and a representation of a typical torque values for each limb separately (bottom part). The grey area represents the analysed timeframe of peak torque for each limb.

A reliable hip dynamometer MuscleBoard [20] was used to bilaterally assess maximal isometric HADD and HABD strength. For each test, participants performed three 5-s isometric repetitions in the supine position with hip, knee and ankle in neutral position. To stabilise the body during testing, the players held on to the sides of the plinths while a non-elastic strap was tightly secured across the pelvis on the anterior side (shown in Fig 2). Force signals (shown in Fig 2) were recorded separately for each leg (at 1000 Hz) and processed with a 10 ms moving average filter using custom-developed software (ARS Dynamometry, S2P, Ljubljana, Slovenia; created in Labview 8.1., National Instruments, Austin, TX, USA). Force values were multiplied by the lever arms (distance from greater trochanter to the contact of the sensor with the lower leg above the ankle) and normalized by the body mass to obtain normalized torque data (Nm/kg). Normalized peak torques of each leg were averaged to obtain one overall value of HADD and HABD strength. Additionally, ADD:ABD ratio and ILA in HADD and HABD strength (%) was calculated using the following equation: (stronger-weaker limb/stronger limb) x 100).

### Statistical analysis

All statistical analyses were performed in SPSS (25.0, SPSS Inc., Chicago, USA). Descriptive statistics were calculated and reported as mean ± standard deviation. Shapiro-Wilk test was used to check normal distribution of variables. Binary logistic regression was performed to assess the impact of 5 predictors (HABD and HADD strength, HABD and HADD ILA, HADD:HABD ratio) on the likelihood that participants would retrospectively report having had groin pain in the past 12 months. Additionally, an independent samples t-test or its non-parametric equivalent Mann-Whitney U test was conducted to assess the differences between the groin pain and the control groups, and between the control—soccer and the control—basketball groups. Where the difference was significant, standardized effect sizes (Cohen’s *d* or *r*) were calculated and interpreted as follows: (0.0−0.2—trivial; 0.2−0.6—moderate; 0.6–1.2—large; >1.2 –very large) [21]. Where applicable, chi-square test for independence (with Yates’ Continuity Correction) was calculated to explore the relationship between categorical variables in the groin pain and the control group. The level of statistical significance was set at p < 0.05.

## Results

The demographics of the players (age, body height, body mass) and the numerus of the different groups are presented in Table 1.

**Table 1:** Demographic data.

Group	Age	Body height [cm]	Body mass [kg]
groin pain—all (n = 27)	17.1 ± 1.5	182.1 ± 8.4	75.9 ± 13.9
groin pain—soccer (n = 20)	17.1 ± 1.6	179.8 ± 6.9	71.8 ± 8.5
groin pain—basketball (n = 7)	17.0 ± 1.1	188.6 ± 9.5	87.5 ± 19.9
control—all (n = 200)	16.9 ± 1.4	184.5 ± 8.5	75.5 ± 11.6
control—soccer (n = 98)	16.8 ± 1.2	180.5 ± 6.8	71.5 ± 9.9
control—basketball (n = 102)	16.9 ± 1.5	188.4 ± 8.2	79.8 ± 11.6

Data is presented as mean ± standard deviation.

### Groin pain

Twenty-seven (11.9%) of all players reported groin pain in the past 12 months. The proportion of players with past year groin pain was statistically significantly different between the sports: 20 (16.9%) in soccer and 7 (6.4%) in basketball (χ^2^(1, n = 227) = 5.03, p = 0.02). Players reported a mean pain intensity of 4.6 ± 1.9. Twelve players (44.4%) reported to be absent from training due to groin pain. Fourteen players (51.8%) had a single episode of groin pain, which lasted on average 18.8 ± 16.1 days, median = 14 days (two players were excluded from calculation of this average because they had groin pain for 144 and 365 days, respectively). Other players had multiple episodes of groin pain throughout the past 12 months: 7 players had fewer than 7 groin pain episodes (17.4 ± 10.8 cumulative days of groin pain) and 6 players had between 12 and 52 groin pain episodes (47 ± 20.3 cumulative days of groin pain).

Maximal isometric HADD and HABD strength, ILA in HADD and HABD strength, ADD:ABD strength ratio and basic information about players’ training in the groin pain and in the control groups are presented in Table 2. The only statistically significant difference between the groin pain group and the control group was found in the age of the players at the start of regular training (7.2 ± 1.8 years vs. 8.5 ± 2.6 years, respectively).

**Table 2:** Strength values and training data for the groin pain and control groups.

	groin pain	control	p values	effect sizes
	(n = 27)	(n = 200)		
HABD [Nm/kg]	1.0 ± 0.1	1.0 ± 0.1	0.29	0.00
HABD ILA [%]	3.1 ± 2.3	3.3 ± 2.9	0.85	0.01
HADD [Nm/kg]	1.1 ± 0.2	1.1 ± 0.2	0.89	0.00
HADD ILA [%]	3.0 ± 2.6	3.6 ± 3.2	0.90	0.01
HADD:HABD ratio	1.03 ± 0.18	1.07 ± 0.17	0.32	0.21
SRT [years]	7.2 ± 1.8	8.5 ± 2.6	0.03*	0.14
Training—weekly frequency	6.6 ± 2.2	6.1 ± 1.9	0.30	0.07
Training duration [min]	98.9 ± 17.2	100.7 ± 18.1	0.73	0.02
Warm up duration [min]	23.6 ± 7.9	21.2 ± 6	0.65	0.03

Data is presented as mean ± standard deviation.

*statistically significant difference; HADD hip adduction; HABD hip abduction; ILA interlimb asymmetry; SRT age at the start of regular training

No statistically significant differences between the groin pain group and the control group were found in the proportion of players with a positive answer regarding training characteristics (shown in Table 3). One hundred thirty-one players (57.7%) reported playing other sports for 2.5 ± 1.6 years prior to playing their main (current) sport: 16 players (59.3%) in the groin pain group for 2.8 ± 1.5 years and 115 players (57.5%) in the control group for 2.5 ± 1.7 years. All players in both groups reported doing stretching during warm-up.

**Table 3:** Data on the proportion of players with a positive answer regarding training characteristics for the groin pain and control groups.

	groin pain	control	χ^2^	p values
	(n = 27)	(n = 200)		
Playing other sports [%]	59.3	57.5	0.0	1.0
Stabilization exercises during warm-up [%]	88.9	71.5	2.86	0.09
Cool down stretching [%]	92.6	86.0	0.42	0.52
Separate resistance training [%]	85.2	79.5	0.19	0.66

χ^2^ chi-square

The logistic regression model with groin pain as the dependent variable and HADD and HABD strength, ILA in HADD and HABD strength, ADD:ABD strength ratio as predictors was not statistically significant χ^2^(5) = 3.59. p = 0.61. The model as a whole explained between 1.6% (Cox and Snell R square) and 3.0% (Nagelkerke R squared) of the variance in groin pain status and correctly classified 88.1% of cases (same as when predictor variables were not included in the model).

### Soccer vs. basketball

Maximal isometric HADD and HABD strength, ILA in HADD and HABD strength, ADD:ABD strength ratio and basic information about players’ training soccer and basketball players are presented in Table 4. Statistically significant differences between groups were found for HADD strength, HADD:HABD ratio, age of the players at the start of regular training, and training duration.

**Table 4:** Strength values and training data for soccer and basketball groups.

	soccer	basketball	p values	effect sizes
	(n = 98)	(n = 102)		
HABD [Nm/kg]	1.0 ± 0.1	1.0 ± 0.1	0.32	0.00
HABD ILA [%]	3.5 ± 2.9	3.2 ± 2.8	0.29	0.07
HADD [Nm/kg]	1.1 ± 0.2	1.0 ± 0.2	0.001*	0.50
HADD ILA [%]	3.4 ± 2.8	3.8 ± 3.5	0.75	0.02
HADD:HABD ratio	1.10 ± 0.18	1.03 ± 0.16	0.007*	0.39
SRT [years]	7.3 ± 2.3	9.6 ± 2.3	<0.001*	0.47
Training weekly frequency	6.0 ± 1.8	6.2 ± 1.9	0.67	0.03
Training duration [min]	94.5 ± 11.1	106.6 ± 21.3	<0.001*	0.34
Warm up duration [min]	21.2 ± 6.6	21.2 ± 5.4	0.36	0.06

Data is presented as mean ± standard deviation.

*statistically significant difference; HADD hip adduction; HABD hip abduction; ILA interlimb asymmetry; SRT age at the start of regular training

Statistically significant difference between soccer and basketball groups were found in the proportion of players with a positive answer regarding the use of stabilization exercises during warm-up, while proportions of players with a positive answer regarding the participation in other sports, the use of cool down stretching and the use of separate resistance training were not statistically significantly different (shown in Table 5). Forty-nine (50.0%) soccer players reported playing other sports for 2.3 ± 1.6 years, while 66 (64.7%) basketball players reported playing other sports for 2.6 ± 1.7 years.

**Table 5:** Data on the proportion of players with a positive answer regarding training characteristics for the soccer and basketball groups.

	soccer	basketball	χ^2^	p values
	(n = 98)	(n = 102)		
Playing other sports [%]	50.0	64.7	3.84	0.06
Stabilization exercises during warm-up [%]	87.8	55.9	23.38	<0.001*
Cool down stretching [%]	87.3	84.7	0.10	0.75
Separate resistance training [%]	79.4	79.6	0.00	1

χ^2^ chi-square

## Discussion

The main purpose of this study was to investigate the differences in several strength and training variables between (a) the group with groin pain in the past year and the control group; (b) the group of youth soccer and basketball players. We have found that: (i) the only significant difference between the groin pain group and the control group is in the age of the players at the start of regular training (7.2 ± 1.8 vs. 8.5 ± 2.6 years, respectively); (ii) the strength variables do not predict whether soccer and basketball players have experienced groin pain in the past year; (iii) soccer players have significantly higher HADD strength and HADD:HABD strength ratio compared to basketball players. In addition, the reference values for HADD and HABD strength, and the HADD:HABD ratio for youth soccer and basketball players are presented.

### Groin pain

The proportion of players who reported groin pain in the past year was 11.9% (16.9% in soccer and 6.4% in basketball), which is lower compared with similar studies that used retrospective methods of self-reported subjective groin pain symptoms (37–49% for adult [16, 22, 23] and 42% for youth soccer players [17]). It is possible that the players in our study already had some experience with prevention training/adductor strengthening and therefore had a lower rate of groin pain, whereas none of the players in the study by DeLang et al. [17] had been exposed to prevention training. Many other different factors could also influence this proportion (differences in age, volume and intensity of training, level of play, honesty/recollection ability when answering questionnaires, etc.). It should be noted that results from retrospective and subjective methods (i.e., self-reported groin pain) may underestimate the true proportion of players with groin pain as only 10% of mild injures are retrospectively remembered [24]. Furthermore, players may also intentionally conceal groin pain to avoid being excluded from team training/competition: 41% of elite soccer players feared that reporting groin pain would result in club medical staff excluding them from playing [17], and 26% of players believed groin pain was normal [25]. Therefore, both subjective and objective measures should be used in secondary prevention, as shown in a study by Wollin et al. [26].

Similarly to other studies [16, 17], our results show no differences between participants with and without groin pain in the past year regarding HADD strength. This confirms the previous findings that (i) lower HADD may not be suggestive of a previous groin injury [27], even though it is well known risk factor for future groin pain [9], and (ii) youth soccer and basketball players do not need to report past year groin pain for the measured HADD strength results to be accurate. The results also indicate that HADD strength of soccer or basketball players can fully recover after the groin pain is reduced, especially if the players continue to engage in activities that require high hip adductors activation [28]. However, Esteve et al. (2018) [16] showed that the duration of groin pain in the past year could be a factor influencing the recovery of HADD strength (adult soccer player who had past year groin pain longer than 6 weeks demonstrated 12–15% lower HADD strength). Similar to the study by DeLang et al. [17] we were unable to confirm whether this is true for youth soccer players due to the small sample size. Players in our study reported that their single episode past year groin pain lasted for 18.8 ± 16.1 days on average. Including players with multiple episodes of groin pain, there were 33.3% of players who had groin pain lasting longer than 6 weeks, of which 6 soccer players (30%), which is a similar to the proportion reported in previous studies (31% in adult [23] and 14% in youth [17] players).

It would be interesting to see if the hip strength results would be similar if only players with adductor-related groin pain were included in the analysis. Namely, the broad definition of groin pain also includes players with hip joint-, hip flexors-, lower abdomen/inguinal- and pubic-related groin pain, according to the DOHA agreement [29]. The listed (sub)types of groin pain do not necessarily affect the HADD strength as much as adductor-related groin pain; further retrospective and prospective studies using a more precise definition of groin pain would be needed to confirm this assumption.

In addition, novel results from our study show that HABD strength, ILA in HADD and HABD strength, and HADD:HABD strength ratio also do not differentiate between youth players with and without past year groin pain. Our results regarding the mean ILA in HADD and HABD isometric strength (3.1–3.8%) is consistent with 3–4% from previous study on elite adult soccer players. However, clinicians should be cautious when measuring ILA in HADD eccentric strength (using break method with hand-held dynamometer or isokinetic dynamometer), as injury-free soccer players displayed 14–22% stronger dominant compared to the non-dominant side [30, 31]. The reason for these differences in ILA between isometric and eccentric HADD strength remains unknown, but it is likely that repetitive kicking causes a specific strength adaptation of the hip adductors, which lengthen during the early swing phase of the kick while being highly active [32, 33].

To our knowledge, this is the first study showing that groin pain risk could be influenced by the age of the players at the start of regular training (7.2 ± 1.8 years for the groin pain group vs. 8.5 ± 2.6 years for the control group). These results further suggest that an early specialization in sport (defined as participating in a single sport, with a deliberate focus on intense training and development in one sport at the exclusion of others) [34] may not be appropriate for children’s optimal physical health, and should be delayed until middle to late adolescence, since it can cause an increased risks of burnout and (overuse) injuries [35–37], such as groin pain. Playing several different sports during childhood or closely monitoring for signs of overuse injuries and burnout are two suitable and recommended alternatives.

### Soccer vs. basketball

The article presents novel results on the (i) the reference values for HADD and HABD strength and HADD:HABD strength ratio in population of youth soccer and basketball players; and (ii) direct comparison of youth soccer and basketball players in aforementioned strength parameters measured by the Muscleboard dynamometer. These results are particularly useful for those involved in injury prevention and (late) rehabilitation in youth soccer or basketball clubs, as they can compare the values of their own players and gain insight into any strength deficiencies which are important for both primary and secondary prevention as well as an indicator of general fitness. Studies have shown that current groin pain significantly reduces HADD strength, both in adult soccer (measured with hand-held dynamometer) [12] and Gaelic football players (measured with sphygmomanometer) [38]. Players with significantly lower HADD strength compared to reference values presented in the present study and other studies [16, 17, 30, 31] would benefit greatly using simple HADD strengthening protocol with Copenhagen adduction exercise, that requires no equipment. This protocol has been shown to (a) elicit high hip adductor activity [39], (b) increase hip adductor strength [40], and (c) reduce the risk of future groin pain [41].

Compared to youth basketball players, youth soccer players have higher HADD strength and consequently a higher HADD:HABD ratio. This is to be expected given the different movement demands of the two sports [42]. Specifically, in soccer, there is a large accumulation of kicks with the inside part of the foot during the training/competition. Such movement cause high muscle force and stress of hip adductors, particularly the adductor longus and gracilis [28]. Kicking a ball from hip extension, external rotation and abduction is also considered to be one of the main mechanisms for the development of (adductor-related) groin pain [3]. Therefore, it is imperative for soccer players to develop and maintain adequate HADD strength (~3.1 Nm/kg) [12] and HADD:HABD ratio (~1.25–1.60) [31] throughout the whole season, as several factors can negatively affect HADD strength (e.g. congested match schedule [43] or insufficient strength training stimulus [44]). Compared to these values, the youth soccer players from our study had lower HADD strength and HADD:HABD strength ratio, most likely due to the different level of play (elite vs. non-elite players) and younger age. Although Beddows et al. [45] have shown that in Dutch hockey, elite players do not necessarily have higher strength values compared to amateur players in lower leagues. On the other hand, several studies have shown a positive relationship between absolute strength and age in youth soccer players [17, 46].

Basketball players reported that their training was slightly longer (94.5 ± 11.1 min vs. 106.6 ± 21.3 min) compared to soccer players. One of the possible reasons for this could be in the greater emphasis on lower-intensity training of technical elements in basketball (e.g., free throw practise), which could increase the training time. Another difference between sports is in the age at the start of regular training: on average, soccer players started playing at significantly younger age than basketball players (median = 7 vs. 10 years old), which suggests that children aged 10 or younger who start (regularly) training soccer as a first sport change sports less frequently than others. The latter is also suggested by the fact that a slightly larger proportion of basketball players (64.7% vs. 50.0% for soccer players) reported playing other sports prior the main (although the difference between the two groups is statistically insignificant; p = 0.06). Finally, a greater proportion of soccer players reported performing stabilization exercises during warm-up. Stabilization exercises are often part of proven exercise based prevention protocols to reduce the proportion of various sports injuries [47, 48]. It appears that the soccer clubs from which the participants in our study were drawn are more aware of the importance of stabilization exercises compared to the basketball clubs. It would be interesting to know the proportion of coaches who are familiar with the most known and established injury prevention program called FIFA 11+, which also includes stabilization exercises.

### Limitations

Some limitations of the study need to be addressed. First and foremost, the possibility of recall bias when using retrospective questionnaires about past injuries cannot be excluded, especially when asking for an estimate of pain severity. We attempted to reduce this by asking a small number of simple questions, clearly defining chronic groin pain, and limiting the time of the groin pain to the last 12 months, as suggested in previous studies [49, 50]. Furthermore, each player had a research assistant available to explain and help them fill in the questionnaire if needed. In addition, a previous study [16] showed that there may be differences in HADD strength between players with past year groin pain who have had symptoms for longer than 6 weeks and others. We were unable to analyse this in our study due to the small sample size of players with such long-lasting symptoms. Another potential limitation is selection bias, as this study was conducted in a convenience sample; caution should therefore be exercised when generalising the results. However, a relatively large cohort of youth soccer and basketball, included in the study gives little reason to believe that using a random sample would have provided different results.

## Conclusion

Hip strength parameters do not differ between youth soccer and basketball players with and without groin pain in the past year, and do not predict whether players have experienced groin pain in the past year. This shows that strength can be accurately measured without players reporting about past (non-current) groin pain. Additionally, players with groin pain in the past year started training regularly at significantly lower age, suggesting the need to monitor the negative consequences (e.g., overuse injuries or burnout) of early sport specialisation. Finally, clinicians should be careful when directly comparing strength between soccer and basketball players, as differences are already apparent in younger populations: soccer players have in general higher HADD and HADD:HABD strength ratio compared to basketball players.
